# Diabetic retinopathy

**Published:** 2011-09

## Book

**Diabetic Retinopathy for the comprehensive ophthalmologist**. Walker J.

This book will be available in electronic format on the new Community Eye Health Update CD, due out with the December 2011 edition of this journal. Look out for your free copy! The book is also available for purchase (US $39.99, soft cover, free delivery) or free download (nine PDF files of 14-59MB each) from http://drcobook.com. Please note that the book is now three years old and that new information has become available, in particular about intravitreal injections. However, the book also covers timeless topics such as informed consent and diabetes control.

## Online resources

**Diabetes grading scheme**: International Clinical Diabetic Retinopathy And Diabetic Macular Edema Disease Severity Scales. International Council of Ophthalmology, October 2002.

http://archive.icoph.org/standards/gdrp.html

**Patient information about diabetic retinopathy** (pdf, 200KB) www.retinalscreening.nhs.uk/userFiles/File/diabeticRetinopathyFacts.pdf

**Patient information about a DR screening programme**: examples in several languages (PDF, 356KB maximum) www.dhsspsni.gov.uk/public_health_diabetic_retinopathy

**Detailed patient information about screening for DR** (pdf, 200KB) www.retinalscreening.nhs.uk/userFiles/File/EyeScreeningForDiabetes.pdf

**Patient information about laser treatment** (pdf, 200KB)

www.retinalscreening.nhs.uk/userFiles/File/PreparingForLaserTreatmentDR.pdf

